# Influence of Molding Parameters on Quasi-Static Mechanical Properties of Al-Rich Al/PTFE/TiH_2_ Active Materials

**DOI:** 10.3390/ma14112750

**Published:** 2021-05-22

**Authors:** Yilei Wang, Chunlan Jiang

**Affiliations:** State Key Laboratory of Explosion Science and Technology, Beijing Institute of Technology, Beijing 100081, China; wylbitDr@163.com

**Keywords:** Al-rich Al/PTFE/TiH_2_ active material, preforming pressure, the pressure holding time, quasi-static compression, microstructure, phase analysis, mechanical properties

## Abstract

Preforming pressure and the pressure holding time are important parameters of the molding process, which directly affect the mechanical properties of materials. In order to obtain the best molding parameters of Al-rich Al/PTFE/TiH_2_ composites, based on the quasi-static compression test, the influence of molding parameters on the mechanical properties of Al-rich Al/PTFE/TiH_2_ composites was analyzed, and the microstructure characteristics of Al-rich Al/PTFE/TiH_2_ specimens were analyzed by SEM. An X-ray diffractometer was used to analyze the phase of the residue after quasi-static compression experiment. The results show that: (1) With the increase in molding parameters (preforming pressure and the pressure holding time), the compressive strength, failure strain and toughness of Al-rich Al/PTFE/TiH_2_ specimens first increase and then decrease. The best molding process parameters of Al-rich Al/PTFE/TiH_2_ materials are preforming pressure 240 MPa and the pressure holding time 100 s. (2) For unsintering specimens, when the preforming pressure is less than 150 MPa, the porosity of the specimen increases slowly at first and then decreases. When the preforming pressure is greater than 150 MPa, the porosity of the specimen increases first and then decreases. When the pressure holding time is no more than 100 s, the porosity of the specimen decreases gradually. When the pressure holding time is more than 100 s, the porosity of the specimen increases first and then decreases. For sintered specimens, when the preforming pressure is less than 100 MPa, the porosity of the specimen decreases gradually. When the preforming pressure is greater than 100 MPa, the porosity of the specimen first increases and then decreases. With the increase in the pressure holding time, the porosity first increases and then decreases. For each preforming pressure specimen, compared with that before sintering, the porosity after sintering either decreases or increases. For each the pressure holding time specimen, the porosity increases after sintering compared with that before sintering. The microstructure of PTFE crystal inside the specimen is mainly planar PTFE crystal. The size and number of planar PTFE crystals are significantly affected by the molding parameters, which further affects the mechanical properties of Al-rich Al/PTFE/TiH_2_ specimens. When the preforming pressure is less than 100 MPa, the planar PTFE crystals are small and few, which results in the worst mechanical properties of the specimens. When the preforming pressure is more than 100 MPa and does not contain 240 Mpa, the planar PTFE crystals are small and there are more of them, which results in better mechanical properties of the specimens. When the preforming pressure is 240 MPa, the planar PTFE crystals are large and numerous, which results in the best mechanical properties of the specimen. When the pressure holding time is 100 s, the planar PTFE crystals are large and there are more of them, which results in the best mechanical properties of the specimen. (3) The reactivity of Al-rich Al/PTFE/TiH_2_ specimens with TiH_2_ the content of 10% under quasi-static compression is not significantly affected by the molding parameters.

## 1. Introduction

Al/PTFE is a typical reaction material, which is impacted at high velocity to produce explosion light and release a lot of chemical energy [1,2]. The reaction materials mainly include thermite, intermetallic compounds, metal polymers, metastable molecular compounds, matrix materials and metal hydrides [3,4,5,6]. Taking Al/PTFE (26.5/73.5 wt%) material as an example, the unit mass energy is 3.5 times that of TNT, and the energy per unit volume is 5 times [7,8] that of TNT, and the chemical energy released is more than 10 times that of TNT kinetic energy [9]. Therefore, the damage elements such as active fragments, liner and warhead shell made of this kind of active material have the characteristics of “impact + reaction”, which obviously increase the damage effect to the target, and the application value in military field is very high.

TiH_2_ is a high energy metal hydride with high hydrogen content. The melting point of TiH_2_ is 400 °C, and above this temperature, TiH_2_ decomposes slowly and dehydrogenates completely at 600–800 °C in vacuum. When the hydrogen content is 3.9%, the calorific value of unit mass released by TiH_2_ is 21.5 MJ/kg [10], which is 2–3 times that of Al/PTFE. The calculated value of Al/PTFE adiabatic reaction temperature is 4306.85 °C, which is enough to completely decompose the TiH_2_ and release a large amount of energy. At present, many scholars have introduced TiH_2_ into conventional energetic materials such as propellants and explosives, and have carried out a series of related properties research. Li [10] studied the effect of TiH_2_ on the burning rate of propellants. It was found that the addition of TiH_2_ can increase the heat of propellant conduction from gas phase to combustion surface, thus increasing the burning rate of propellant. Cheng [11] added TiH_2_ to the emulsion explosive for underwater explosion experiment. The experimental results show that the total energy and specific impulse of the mixed explosive are obviously improved, and the explosive ferocity is higher than that of the emulsion explosive without TiH_2_. Some scholars [12,13,14] introduced TiH_2_ into the strong oxidant KClO_4_, and studied the compatibility and critical ignition temperature of the mixture. It was found that the decomposition of TiH_2_ in the strong oxidant was negligible, and the ignition temperature of KClO_4_ would not change due to the addition of TiH_2_.

Molding process is an important link in the preparation of material specimens, which directly affects the properties of material specimens. In the last decade, scholars have mainly focused on researching the molding process of non-active materials, and there are not many reports on this kind of research. Zhu [15] studied the influence of molding process conditions on the properties of MCMB/graphite composites, and analyzed the influence of different molding pressure on the resistivity, bending strength and compressive strength of MCMB/graphite composites, and reporting that the resistivity and mechanical properties of MCMB/graphite composites are mutually exclusive, and the molding pressure of MCMB/graphite composite bipolar plate is 3–4 MPa. Cui [16] studied the effect of molding pressure on the mechanical properties of PES resin material. The results showed that the tensile properties and tensile modulus of the modified PES resin material were the best when the vacuum pressure was 0.25 MPa. Ma [17] studied the influence of molding process parameters on the mechanical properties of thermoplastic PF/CF composites, discussed the influence of the pressure holding time on the mechanical properties of composites, and determined that the thermoplastic with chopped CF mass fraction in the range of 5–25%, and the optimal the pressure holding time of PF/CF composite molding, was 15 min. Hu [18] prepared 3D-SiC_f_/ZL301 composite with 48% fiber volume fraction by vacuum assisted pressure infiltration method, and studied the effect of infiltration holding pressure time on Microstructure and mechanical properties of 3D-SiC_f_/Al composite. The results show that prolonging the pressure holding time can reduce the void and agglomeration in the composite fiber bundle, and the tensile strength of the composites increases first and then decreases with the increase in holding pressure time, which is due to the deterioration of the mechanical properties of the composites due to the strong interfacial reaction. Xia [19] Studied the influence of molding pressure on the water absorption and bulk density of samples. The results show that under the above conditions, when the molding pressure is 140 MPa, the water absorption and bulk density of Al_2_O_3_-TiC/Fe matrix composite ceramics reach the best value, the hardness is 467 HB, the bending strength is 882 MPa, the relative density is 99.6%, and the wear rate is 0.102%. 

Recently, Yu [20,21] introduced TiH_2_ into Al/PTFE energetic materials for the first time under zero oxygen balance, and mainly aimed to analyze the effect of different TiH_2_ content on the static mechanical properties of Al/PTFE/TiH_2_ composites under equilibrium condition. Through quasi-static compression experiments, the effects of different content of TiH_2_ on the mechanical properties and reaction characteristics of Al/PTFE were compared and analyzed. The results showed that when the content of TiH_2_ was 5%, the probability of reaction was 90% and the material strength reached the maximum value of 108 MPa, which was 15.1% higher than that of Al/PTFE material. Although the content of TiH_2_ has a significant effect on the mechanical properties and reaction characteristics of Al/PTFE active material, the jet formed by the Al/PTFE active cover under zero oxygen balance is easy to diverge, which is not conducive to the penetration and perforation of the target, while excessive Al can enhance the cohesiveness of the Al/PTFE active jet, thus improving the penetration effect of the target. At the same time, Yu did not study the effect of technology on the properties of Al/PTFE/TiH_2_ composites under equilibrium. Therefore, the preparation process, mechanical properties and reaction characteristics of Al-rich Al/PTFE/TiH_2_ active composites composed by adding TiH_2_ into Al/PTFE energetic materials with excess Al are still unclear. At present, no research papers on the technology of Al/PTFE/TiH_2_ active materials under equilibrium have been published at home and abroad. Based on the above, this paper starts from the molding process of the preparation process, the influence of six groups of preforming pressure parameter (60 MPa, 100 MPa, 150 MPa, 200 MPa, 240 MPa, and 280 MPa) and four groups of the pressure holding time parameter (50 s, 100 s, 150 s, and 200 s) on the mechanical properties and micromorphology of Al-rich Al/PTFE/TiH_2_ materials were analyzed by quasi-static compression experiments.

## 2. Materials and Methods

### 2.1. Materials

The main raw material powders are as follows: Polytetrafluoroethylene (PTFE): 27 μm (from 3M, Shanghai, China); Al: 6–7 μm (from JT, Dalian, China); TiH_2_: 4–6 μm (from RF, Zhuzhou, China); Anhydrous ethanol: purity 95% (from TG, Beijing, China). The physical and chemical properties of each component are listed in Table 1.

### 2.2. Sample Preparation

According to the main reaction equation of the material: 4Al + 3C_2_F_4_ = 4AlF_3_ + 6C, it can be seen that when TiH_2_ is introduced into Al-rich Al/PTFE (wt50%/wt50%) active material, the maximum content of TiH_2_ is 32%. According to the reference [20,21], the Al/PTFE/TiH_2_ active material with TiH_2_ content of 10% has moderate energy release rate and reaction threshold. Therefore, In this paper, Al (wt40%)/PTFE (wt50%)/TiH_2_ (wt10%) was selected as the composition of 10 groups of specimens.

Assuming that the volume fraction of PTFE powder is $V_{PTFE}$, the volume fraction of Al powder is $V_{Al}$, and the volume fraction of TiH_2_ is $V_{TiH_{2}}$, the total mass of Al-rich Al/PTFE/TiH_2_ mixed powder is as follows:

(1) $$m=\rho_{TMD}\left(V_{Al}+V_{PTFE}+V_{TiH_{2}}\right)$$

Assuming that the mass fraction of Al powder is $w_{Al}$, the density of Al powder is $\rho_{Al}$, the mass fraction of PTFE is $w_{PTFE}$, the density of PTFE powder is $\rho_{PTFE}$, the mass fraction of TiH_2_ is $w_{TiH_{2}}$, and the density of TiH_2_ is $\rho_{TiH_{2}}$, then

(2) $$\left\{ \begin{array}{l} V_{Al}=\frac{w_{Al}\times m}{\rho_{Al}}\\ V_{PTFE}=\frac{w_{PTFE}\times m}{\rho_{PTFE}}\\ V_{TiH_{2}}=\frac{w_{TiH_{2}}\times m}{\rho_{TiH_{2}}} \end{array}\right.$$

According to Equations (1) and (2), the maximum theoretical density of Al-rich Al/PTFE/TiH_2_ is as follows:

(3) $$\rho_{TMD}=\frac{\rho_{Al}\rho_{PTFE}\rho_{TiH_{2}}}{w_{Al}\rho_{PTFE}\rho_{TiH_{2}}+w_{PTFE}\rho_{Al}\rho_{TiH_{2}}+w_{TiH_{2}}\rho_{Al}\rho_{PTFE}}$$

Assuming that the mass of the pending specimen in the air is m_1_, pending specimen is suspended by a gravimeter and placed in a beaker with water and does not touch the wall of the beaker, the measured gravity is N, the volume of the pending specimen is V, the corresponding density of water at ambient temperature is ρ_water_, and the local acceleration of gravity is g_local_. According to Archimedes’ principle [22], then

(4) $$\rho_{water}g_{local}V=m_{1}g_{local}-N$$

namely,

(5) $$V=\frac{m_{1}g_{local}-N}{\rho_{water}g_{local}}$$

Combined with Equations (4) and (5), the actual density of the specimen is as follows:

(6) $$\rho_{true}=\frac{m_{1}}{V}=\frac{m_{1}}{\frac{m_{1}g_{local}-N}{\rho_{water}g_{local}}}=\frac{m_{1}}{m_{1}g_{local}-N}\rho_{water}g_{local}$$

**Figure 1:**
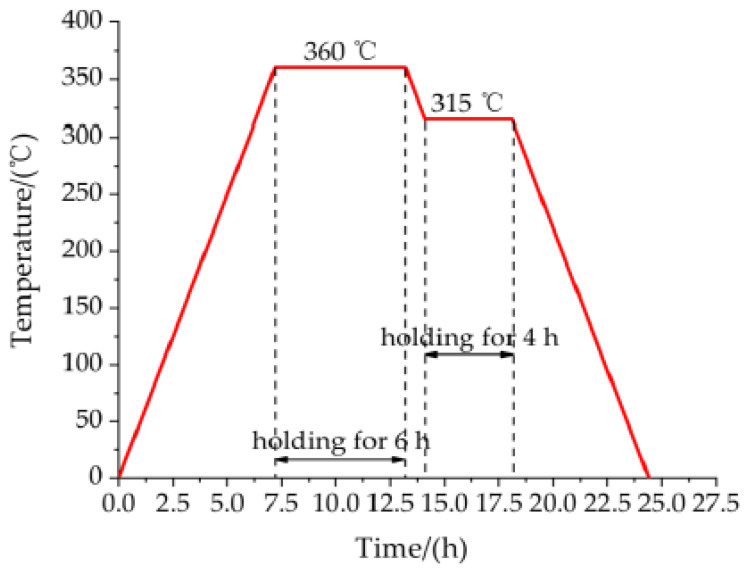
Sintering control curve of experimental specimen.

**Figure 2:**
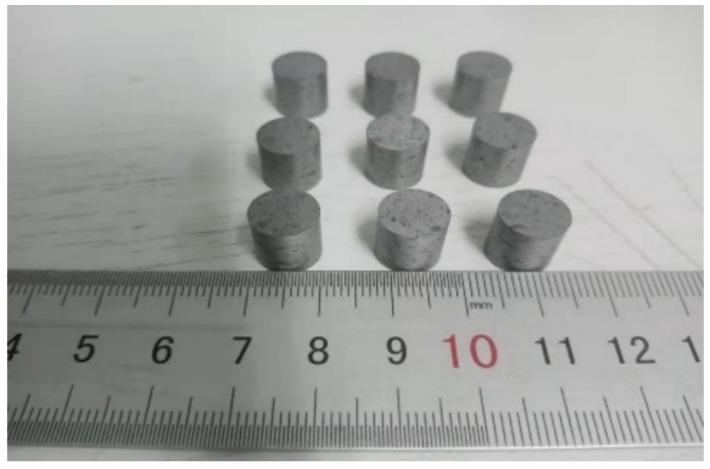
Physical drawings of some Al-rich Al/PTFE/TiH_2_ experimental specimens.

The complete process of preparing Al-rich Al/PTFE/TiH_2_ active specimens is as follows:

1. Drying of mixture: according to the mass ratio of 40/50/10, Al powder, PTFE powder and TiH_2_ powder are weighed by electronic scale and mixed in a beaker. While adding an appropriate amount of anhydrous ethanol into the beaker, the mixture is continuously stirred for about 30 min to make a “sesame paste”-like liquid, that is, the three materials are fully mixed. The mixed liquid was dried in a vacuum drying oven at 55 °C for 48 h, and the bulk Al-rich Al/PTFE/TiH_2_ solid mixture was obtained.
2. Compaction: the block Al/PTFE/TiH_2_ solid mixture is crushed with a glass rod, and continuously stirred to powder state, then put into a transparent bag, and then drop the transparent bag containing powder for at least 30 times to obtain uniform Al-rich Al/PTFE/TiH_2_ powder. The powder is pressed into cylindrical large cake specimens with different preforming pressure and holding time by hydraulic press and forming mold.
3. Sintering process: in the sintering furnace with argon atmosphere, evenly raise the temperature of columnar large round cake to 360 °C at the rate of 50 °C/h, holding for 6 h, then evenly lower the temperature to 315 °C at the rate of 50 °C/h, holding for 4 h, and then uniformly lower the temperature to room temperature at the rate of 50 °C/h. The sintering process curve is shown in Figure 1. Take out the large round cake specimen and place it in room temperature for 2 days to eliminate the internal stress. Finally, the Φ 10 mm × 10 mm Al-rich Al/PTFE/TiH_2_ specimen is obtained by machining. Some of the experimental specimens are shown in Figure 2.

### 2.3. Experimental Procedures

**Figure 3:**
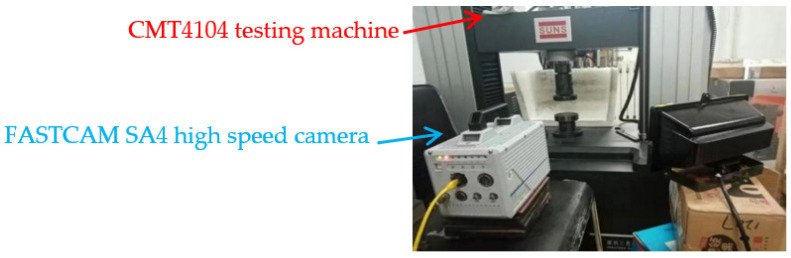
Layout of quasi static compression experiment.

The experimental arrangement is shown in Figure 3. The quasi-static compression test is carried out on 9 groups of experimental specimens by using CMT4104 microcomputer controlled electronic universal testing machine. The experimental loading strain rate $\dot{\varepsilon }$ is 0.1 /s, the corresponding pressure head uniform pressing rate is 60 mm/min, the maximum experimental force is 50 kN, the accuracy level is 0.5, the voltage is 220 V, and the power is 0.4 kW. In order to obtain stable and reliable data, at least three repeated experiments were carried out on each group of experimental specimens. Before the experiment, in order to reduce the friction between the indenter and the end of the specimen and help the transverse deformation of the end face of the specimen, a proper amount of Vaseline was applied on the end face of the specimen. All experiments were carried out at room temperature. The compression process of the test specimen was recorded by FASTCAM SA4 high-speed camera. The shooting speed is 50 fps, the resolution is 512 × 512 ppi, and the starting trigger point is used to capture. Scanning electron microscope (SEM) of FEINOVA450 (FEI, New York, NY, USA) was used to analyze the microstructure of the bottom surface of the specimen.

The X-ray diffractometer (XRD) of Rigaku smartlab 9 (Rigaku, Tokyo, Japan) was used for phase analysis of the sintered specimens. The instrument parameters were set as follows: The tube voltage was 40 kV, the current was 150 mA, Cu-k_α_ radiation λ = 0.15416 nm), the scanning range 2θ was 10–90°, the scanning step was 0.02°, and the scanning speed was 4°/ min.

## 3. Results and Discussion

### 3.1. Microstructure Analysis

**Figure 4:**
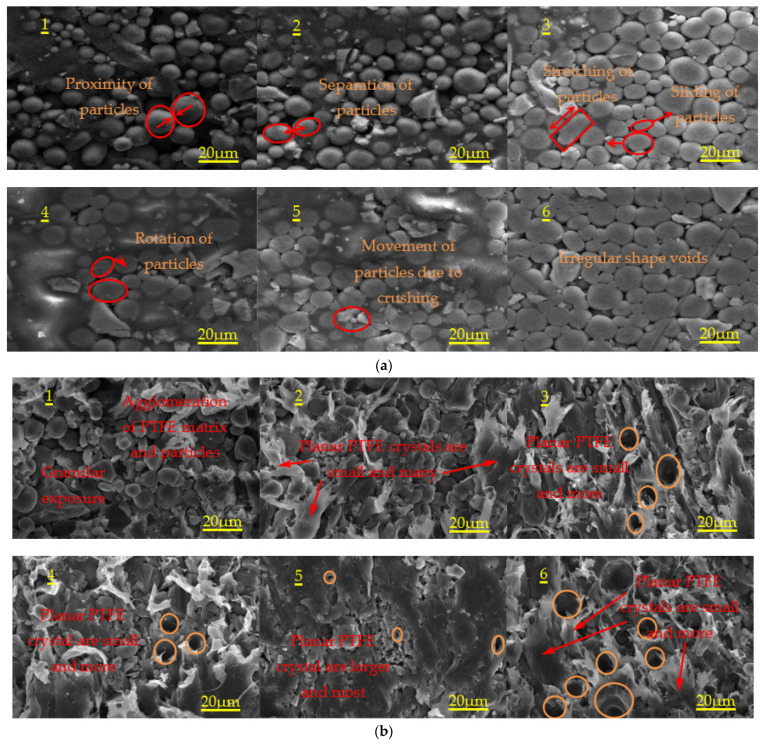

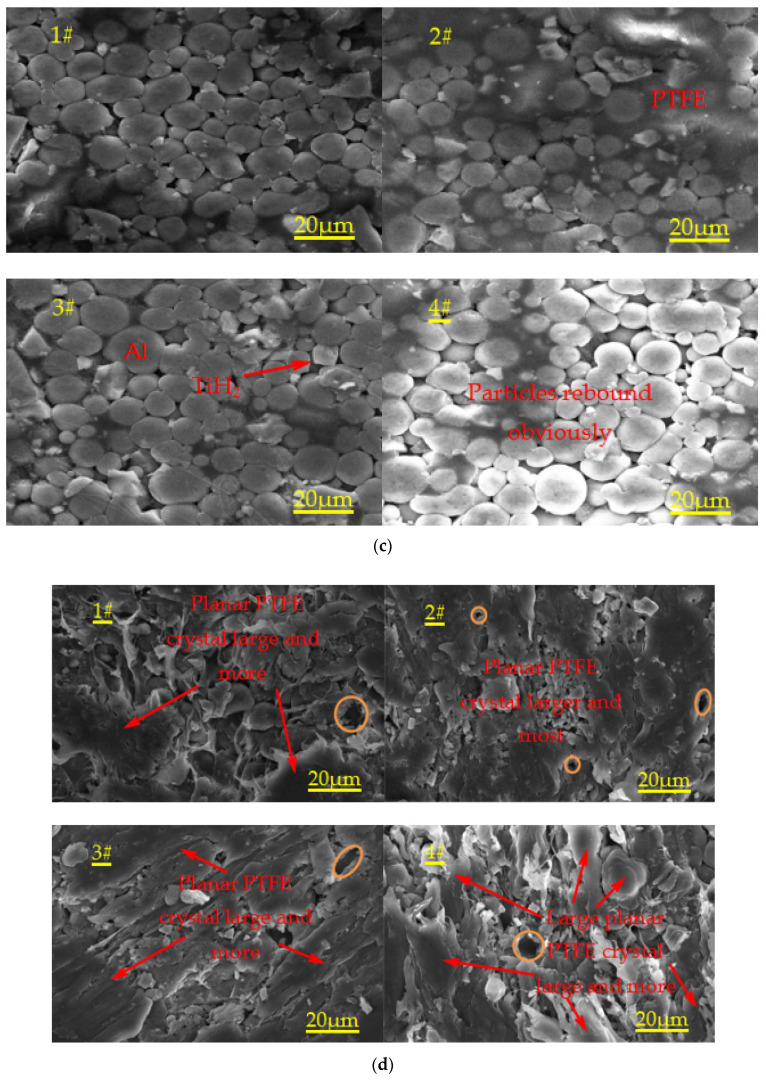
SEM images of specimens before and after sintering under different preforming pressure and the pressure holding time. (**a**) Microstructure of specimens 1, 2, 3, 4, 5 and 6 before sintering; (**b**) Microstructure of specimens 1, 2, 3, 4, 5 and 6 after sintering; (**c**) Microstructure of specimens 1#,2#,3# and 4# before sintering; (**d**) Microstructure of specimens 1#,2#,3# and 4# after sintering.

In order to observe the microstructure changes of the experimental specimen before and after sintering under different preforming pressure and the pressure holding time, the microstructure of the specimens before and after sintering was analyzed by means of scanning electron microscope (SEM). Figure 4a,b show the SEM images of Al-rich Al/PTFE/TiH_2_ active specimens before and after sintering under different preforming pressures, and Figure 4c,d show the SEM images of specimens before and after sintering under different the pressure holding time. Chocolate yellow circle in Figure 4 represents the voids between the particles in the specimen. In Figure 4, the red font and the red circle represent the relevant interpretation and the particle displacement form, respectively. According to the change of these voids and the deformation state of the particles, the microscopic appearance rule of the specimen under the molding parameters can be obtained.

**Figure 5:**
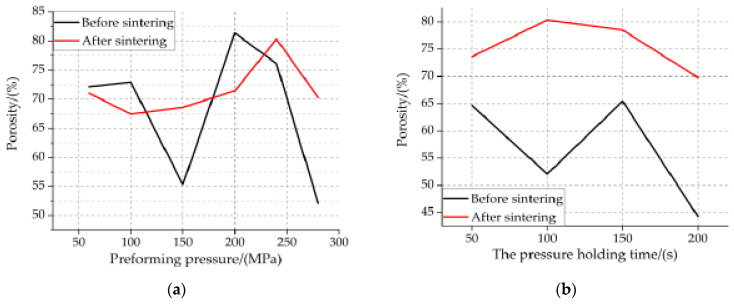
Variation Trend of porosity of Al-rich Al/PTFE/TiH_2_ specimens before and after sintering under molding parameters. (**a**) variation trend of porosity under different preforming pressures; (**b**) Variation trend of porosity under different the pressure holding time.

The SEM of Figure 4 was measured by image analysis software, thus the porosity of specimens under different molding parameters was obtained. The porosity of samples before and after sintering under different molding parameters is shown in Figure 5.

Combined with Figure 4 and Figure 5, it can be concluded as follows:

1. Unsintered: with the increase in preforming pressure, the deformation of particles gradually changed from the initial point contact to the surface contact, the contact area increases gradually, the interface between particles increases gradually, the sliding and deformation of grain boundary between particles decrease gradually, and the shape and orientation of voids are very irregular. Al particles change from spherical to flat with irregular orientation. From large irregular space to small irregular space. Because of the irregularity of TiH_2_ particles, not only the interface between TiH_2_ particles and Al particles and between Al and Al particles increases gradually, but also there are voids at the interface boundary. For each preformed pressure specimen, it has the same particle movement mode under external force loading, as shown in Figure 4a.
  It can be seen from Figure 5a,b that, when the preforming pressure is less than 150 MPa, the porosity of the specimen first increases slowly and then decreases; when the preforming pressure is greater than 150 MPa, the porosity of the specimen first increases and then decreases. This phenomenon may be due to the reduction in the number and volume of voids inside the specimen when the preforming pressure is less than 150 MPa, resulting in the decrease in porosity. When the preforming pressure is greater than 150 MPa, the particles inside the specimen deform, resulting in the increase in the voids between the particles, thus increasing the porosity of the specimen. When the preforming pressure is not less than 200 MPa, the internal stress between particles begins to impede the further deformation of particles, resulting in the gradual decrease in interparticle voids, thus the porosity of the specimen begins to decrease. When the pressure holding time is less than 100 s, the porosity of the specimen decreases gradually. When the pressure holding time is greater than 100 s, the porosity of the specimen increases first and then decreases. This phenomenon may be due to the result of joint action of void exclusion and elastic internal stress. According to Figure 4c,b, with the increase in the pressure holding time, the total volume of internal voids of specimen 3 is the largest, followed by that of specimen 1, and that of specimen 4 is the smallest.
2. Sintered: It can be seen from Figure 5a,b that when the preforming pressure is less than 100 MPa, the porosity of the specimen decreases gradually. When the preforming pressure is greater than 100 MPa, the porosity of the specimen first increases and then decreases. With the increase in the pressure holding time, the porosity first increases and then decreases. For each preforming pressure specimen, compared with that before sintering, the porosity after sintering either decreases or increases. For each the pressure holding time specimen, the porosity increases after sintering compared with that before sintering. It shows that the elastic aftereffect and shrinkage phenomenon of the samples with different preforming pressure after sintering occurred. The elastic aftereffect phenomenon of the samples with different the pressure holding time after sintering occurred.
  It can be seen from Figure 4b,d that the main structure of recrystallized PTFE is planar PTFE crystal. The planar PTFE crystals are large and numerous, and the PTFE matrix and the particles inside the specimen are most closely bonded, and the approximate spherical void is few and small, and then the interface strength of particles is the highest, thus the mechanical properties of the specimens are the best; The planar PTFE crystals are small and numerous or the planar PTFE crystals are large and few, and the PTFE matrix inside the specimen is well bonded with the particles, and the voids are more and larger, and then the interface strength of the particles is higher, thus the mechanical properties of the specimens are better; The planar PTFE crystals are small and few, and the PTFE matrix and particle aggregates are more, and the particles are exposed, and the PTFE matrix and particles are not tightly combined, the voids are more and larger, and then the interface strength of particles is the weakest, thus the mechanical properties of the specimen are the worst. It can be seen from Figure 4b that the internal planar PTFE crystal of specimen 1 is small and few, so the overall mechanical properties of specimen 1 are the worst; The internal planar PTFE crystals inside the specimens 2, 3, 4 and 6 are small and numerous; however, the number of planar PTFE crystals inside the specimens 3 and 6 is obviously more than that inside the specimens 2 and 4. Therefore, the overall mechanical properties of specimens 3 and 6 are better than those of specimens 2 and 4; The number of planar PTFE crystals inside the specimen 3 is more than that inside the specimen 6, and the number of voids inside the specimen 6 is more than that inside the specimen 3. Therefore, the mechanical properties of specimen 3 are better than that of specimen 6; the internal planar PTFE crystals inside the specimen 5 are large and numerous; therefore, specimen 5 has the best mechanical properties. It can be seen from Figure 4d that there are planar PTFE crystals inside the specimens 1#, 2#, 3# and 4#, and the planar PTFE crystals inside the specimen 2# are large and numerous, and the number of planar PTFE crystals inside the specimen 1# is higher than that inside the specimen 3#, and the number of planar PTFE crystals inside the specimen 3# is higher than that inside the specimen 4#. Therefore, the mechanical properties of specimen 2# are the best, and the mechanical properties of specimen 4# are the worst.

### 3.2. Analysis of Static Pressure Mechanical Properties of Active Materials under Molding Parameters

**Figure 6:**
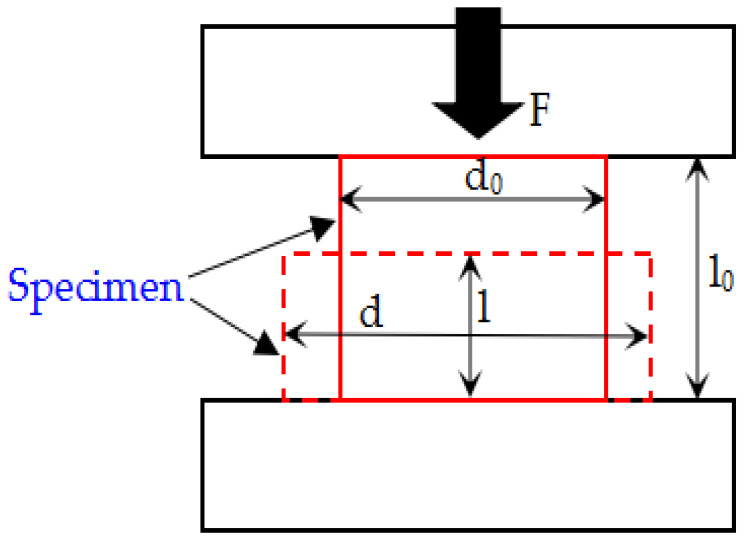
Sketch of loading of quasi-static compression specimens.

In order to facilitate the transformation between the engineering stress and strain and the real stress and strain, it can be considered that all experimental specimens always keep the cylindrical shape and volume unchanged during the compression process, as shown in Figure 6.

The original diameter of the specimen is d_0_, l_0_ is the initial height of the specimen, l is the instantaneous height of the specimen; F is the instantaneous loading stress measured by the universal material testing machine, S is the instantaneous area of the specimen, d is the instantaneous diameter of the specimen, The formula is as follows:

(7) $$\begin{array}{l} \pi {\left(\frac{d_{0}}{2}\right)}^{2}l_{0}=Sl\\ \left\{ \begin{array}{l} \sigma_{eng}=\frac{F}{\pi {\left(\frac{d_{0}}{2}\right)}^{2}}=\frac{4F}{\pi d_{0}^{2}}\\ \varepsilon_{eng}=\frac{l_{0}-l}{l_{0}}=1-\frac{l}{l_{0}} \end{array}\right. \end{array}$$

where σ_eng_ is the engineering stress and ε_eng_ is the engineering strain. The following formulas of true strain (ε_true_) and true stress (σ_true_) can be obtained by combining with the above Equation (7).

(8) $$\left\{ \begin{array}{l} \varepsilon_{true}=\int_{l}^{l_{0}}d\varepsilon =\int_{l}^{l_{0}}\frac{dl}{l}=\ln\frac{l_{0}}{l}=\ln\frac{1}{1-\varepsilon_{eng}}=-\ln\left(1-\varepsilon_{eng}\right)\\ \sigma_{true}=\frac{F}{S}=\frac{Fl}{\pi {\left(\frac{d_{0}}{2}\right)}^{2}l_{0}}=\frac{\frac{\pi d_{0}^{2}\sigma_{eng}}{4}l}{\pi {\left(\frac{d_{0}}{2}\right)}^{2}l_{0}}=\frac{\sigma_{eng}l}{l_{0}}=\sigma_{eng}\left(1-\varepsilon_{eng}\right) \end{array}\right.$$

#### 3.2.1. Influence of Preforming Pressure

As shown in Table 2, six groups of Al-rich Al/PTFE/TiH_2_ specimens were prepared under different preforming pressures.

It can be seen from Table 2 that when the holding time is fixed, the density of the active material increases with the increase in the preforming pressure. When the preforming pressure increases to 240 MPa, the density of the active material increases slowly. The analysis shows that as the preforming pressure continues to increase, the displacement between particles has been greatly reduced, and the deformation between particles has not yet begun. When the preforming pressure exceeds the critical stress of particles, the particles begin to deform, and the density of the specimen continues to increase. When the preforming pressure increases to a certain extent, the work hardening caused by the severe deformation of the particles makes it difficult to further deform the particles [23]. The density of the specimen increases rapidly from 60 MPa to 240 MPa. This is because the particles occur displace and fill the pores, so when the pressure increases slightly, the density of the specimen increases rapidly [24]. When the preforming pressure is higher than 240 MPa (including 240 MPa), the density of the specimen is almost unchanged, reaching 97.510% of the maximum theoretical density (TMD).

**Figure 7:**
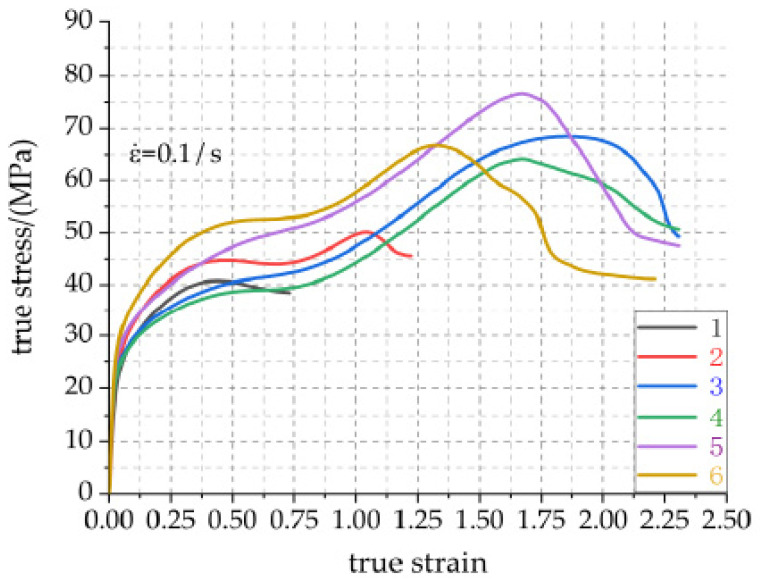
Real stress–strain curves of Al-rich Al/PTFE/TiH_2_ specimens 1, 2, 3, 4, 5 and 6 under different preforming pressures.

Combined with the above Equation (8), the visualization curve of the real stress–strain data of each group of experimental specimens 1, 2, 3, 4, 5 and 6 is given in Figure 7.

It can be seen from Figure 7 that Al-rich Al/PTFE/TiH_2_ active material is a kind of typical strong and tough aluminum polytetrafluoroethyl composite, and its stress–strain curve can be divided into three stages: elastic deformation stage, strain hardening stage, failure stage of strain softening fracture [25]. Its specific characteristics are: in the stage of elastic deformation, the ratio of stress to strain is a constant, that is, the elastic modulus. At this stage, the particles in the specimen are within the elastic limit. With the increase in strain, when the stress exceeds the elastic limit, the material yields and the elastic deformation stage ends; In the strain hardening stage, after the material particles yield and slip, with the increase in stress, the particles inside the specimen are squeezed and twisted along the direction of the maximum stress, thus improving the compressive strength of the material. At this stage, the metal particles bear most of the stress, which is specifically manifested as the metal particles form a force chain [26] to bear this part of the stress. At the end of strain hardening, the PTFE matrix reaches the strength limit and enters the softening stage. With the increase in stress, the deformation rate of the material increases. At this stage, due to greater strain, the specimen is easy to form shear cracks and fracture failure occurs. It may be that the physical crosslinking points of PTFE matrix macromolecular chain are recombined to form supramolecular structure which is favorable for crack development.

Table 3 shows the compression mechanical properties of Al-rich Al/PTFE/TiH_2_ active materials under different preforming pressures. The physical meaning of material toughness in the table indicates the energy absorbed per unit volume of specimen before fracture failure. The better the toughness, the higher the energy absorbed by the specimen, and the stronger the fracture resistance of the specimen. The visualized curves of real stress–strain data of active materials are greatly influenced by preforming pressure as a whole. It can be seen from Figure 7 that in the elastic stage, the elastic modulus of sample 1 is obviously less than that of sample 2, and the elastic modulus of sample 2 is less than that of specimens 3, 4, 5 and 6, while the elastic modulus of specimens 3, 4, 5 and 6 is not much different. Because of the small preform pressure and the small compactness between PTFE matrix particles, specimen 1 is prone to shear deformation and fracture failure under large load. Specimens 2, 3, 4, 5 and 6 are all experienced elastic deformation, yield, strain hardening, strain softening fracture failure process [27]. Especially in the strain hardening and failure stage of strain softening fracture, the stress–strain curves of the active materials were significantly different under different preforming pressures, which may be due to the different contact tightness between PTFE matrix and particles in different pressure specimens. The differences are reflected in the main mechanical property parameters given in Table 3. It can be seen from Table 3 that the maximum compressive strength of the active specimen is 76.672 MPa when the preforming pressure is 240 MPa, and the minimum compressive strength is 40.777 MPa when the preforming pressure is 60 MPa; the maximum toughness of the specimen is 92.213 MJ/m^3^ when the preforming pressure is 150 MPa, which is 2.8% higher than that of the specimen with the preforming pressure of 240 MPa, but its strength is slightly lower. Based on the above analysis, the material has high strength and strong fracture resistance when the preforming pressure is 240 MPa and the pressure holding time is 100 s.

According to the formula of experimental mass loss rate, the strength of specimen with different preforming pressure and the pressure holding time can be characterized. The original mass M of the sample is 1.956 g and the residue mass m g. the formula of the experimental mass loss rate η [28] is as follows:

(9) $$\eta =\frac{M-m}{M}$$

According to the measured residual mass m, the mass loss rate of the specimen 1, 2, 3, 4, 5 and 6 can be obtained by using Equation (9), which is listed in Table 4.

According to Table 4, the experimental mass loss rate of residue 5 is the smallest, which is 4.652%, and then the strength of residue 5 is the best. The experimental mass loss rate of residue 1 is the largest, which is 10.532%, and then the strength of residue 1 is the worst. The data change trend of the experimental mass loss rate is consistent with the change trend of compression strength of specimens in Table 3. It is shown that the theoretical calculation under different preforming pressures is in good agreement with the experimental data. 

#### 3.2.2. Influence of the Pressure Holding Time

According to Section 3.2.1, the strength and toughness of Al-rich Al/PTFE/TiH_2_ active material are the best when the preforming pressure is 240 MPa and the pressure holding time is 100 s. On this basis, the pressure holding time of 50 s, 150 s and 200 s was set, and the quasi-static compression experiment was carried out on the active material specimen. The specific active material parameters are shown in Table 5.

It can be seen from Table 5 that the material density decreases slowly with the increase in the pressure holding time, which is opposite to the gradual increase in material density within a certain time range under a certain pressure. It can be deduced that when the pressure holding time is less than 50 s, with the increase in the pressure holding time, the specimen density may increase gradually, and it may increase first and then decrease. The above phenomena may be caused by the following reasons: (1) the pressure transmission of the press is not enough, which is not conducive to the homogenization of the density of each part of the specimen; (2) The air in the voids between particles is not fully removed; (3) The mutual engagement and deformation between particles are not enough; (4) In the process of pressing, the height of each part of the specimen is uneven and there are errors in height measurement. So far, it can be considered that the maximum density of Al-rich Al/PTFE/TiH_2_ specimen is 2.430 g/cm^3^ and 97.590% of the maximum theoretical density (TMD) when the pressure holding time is 50 s and the preforming pressure is 240 MPa.

**Figure 8:**
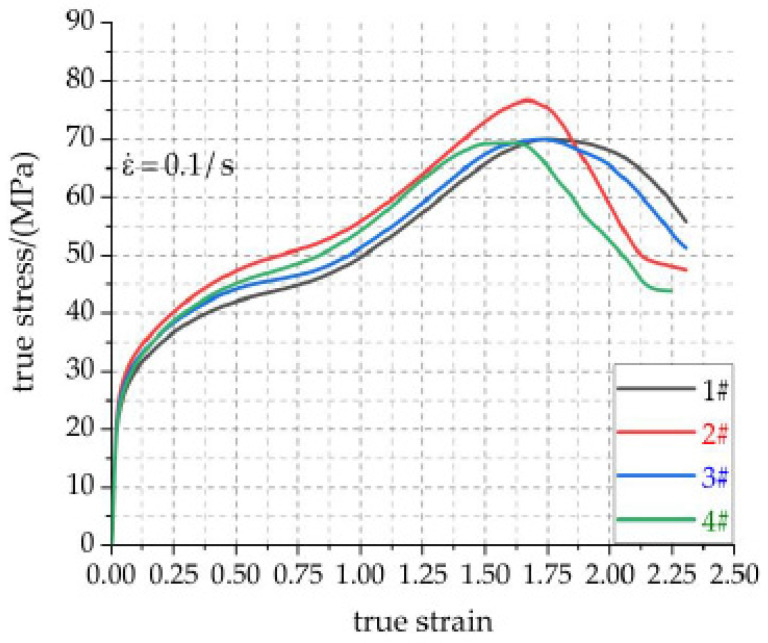
Real stress–strain curves of active material specimens 1#, 2#, 3# and 4# under different holding time.

**Figure 9:**
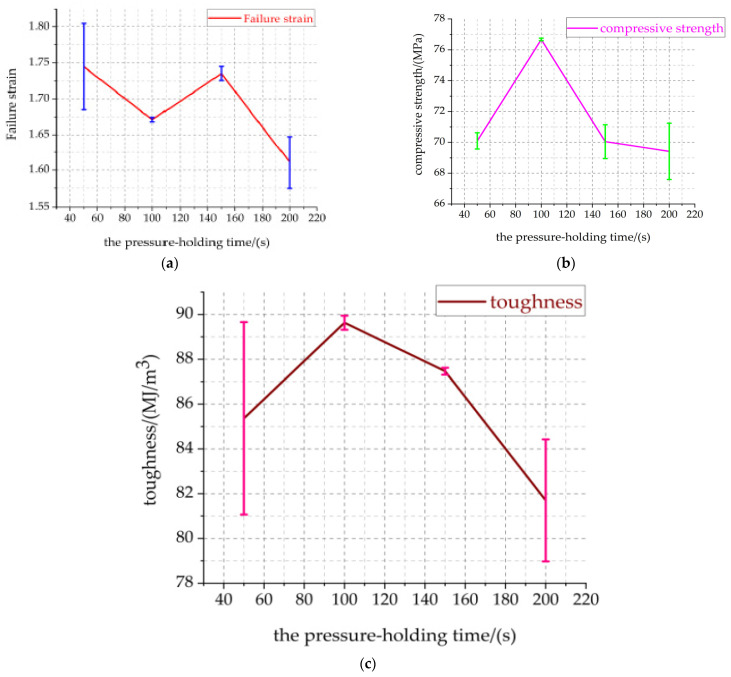
Relationship between compressive strength, failure strain, toughness and the pressure holding time. (**a**) Failure strain—the pressure holding time (**b**) Compressive strength—the pressure holding time. (**c**) Toughness—the pressure holding time.

Combined with the above Equation (8), the real stress–strain data visualization curves of Al-rich Al/PTFE/TiH_2_ specimens under different holding time are shown in Figure 8. Figure 9 shows the relationship between the pressure holding time and compressive mechanical properties of active materials.

Figure 8 shows that, in the elastic stage, the elastic modulus of the four groups of specimens is basically the same, which is not affected by the pressure holding time. In the strain hardening stage and the strain fracture failure stage, the real stress–strain curves of the specimens are significantly different, especially the main mechanical parameters such as compressive strength, failure strain and toughness are significantly affected by the pressure holding time. It can be seen from Figure 9a that the failure strain of the specimen decreases with the increase in the pressure holding time. The compressive strength and toughness first increase and then decrease with the increase in the pressure holding time, as shown in Figure 9b,c. The maximum true strain of specimens 1#, 2#, 3# and 4# is greater than 2.125, indicating that the specimens with different the pressure holding time have good ductility. The failure strain of the specimen 1# reaches to a maximum of 1.7447. The compressive strength and toughness of the specimens 2# are 76.672 MPa and 89.630 MJ/m^3^, respectively. In the deformation of the specimens with different the pressure holding times, the deformation of the specimen 4# is the smallest and the failure is the fastest. The density, compressive strength and toughness of the specimen 4# are the lowest, which are 2.397 g/cm^3^, 69.415 MPa and 81.700 MJ/m^3^, respectively. It may be that the density uniformity of specimen 4# is the worst during the pressing process. It is believed that the property of the material is decreased due to the fact that the PTFE matrix is not tightly bonded with the particles and the PTFE matrix is not completely coated with particles. The material with high strength and good toughness can be used in the warhead damage element to enhance the penetration effect on the target. According to Figure 9a, the failure strain error of specimen 2# is the smallest, and that of specimen 1# is the largest. According to Figure 9b, the material strength error of specimen 2# is the smallest, and that of specimen 4# is the largest. According to Figure 9c, the toughness error of specimen 3# is the smallest, followed by that of specimen 2#, and that of specimen 1# is the largest. Based on the above analysis, specimen 2# has the largest strength, the best toughness, stability and reliability.

According to Equation (9), the mass loss rate of specimens 1#, 2#, 3# and 4# can be obtained, as shown in Table 6.

It can be seen from Table 6 that with the increase in holding time, the experimental mass loss rate of the specimens first decreases and then increases, which is similar to the change trend of compressive strength obtained by theoretical calculation. The experimental mass loss rate of the specimen 2# is the smallest, which is 4.652%, and its strength is the best. The experimental mass loss rate of the specimen 4# is the largest, which is 17.791%, and its strength is the worst. It is shown that the experimental data agree well with the theoretical calculation under different the pressure holding time.

### 3.3. Discussion on Compression Process of Specimen and Phase Analysis of residue After Quasi-Static Compression Experiment

**Figure 10:**
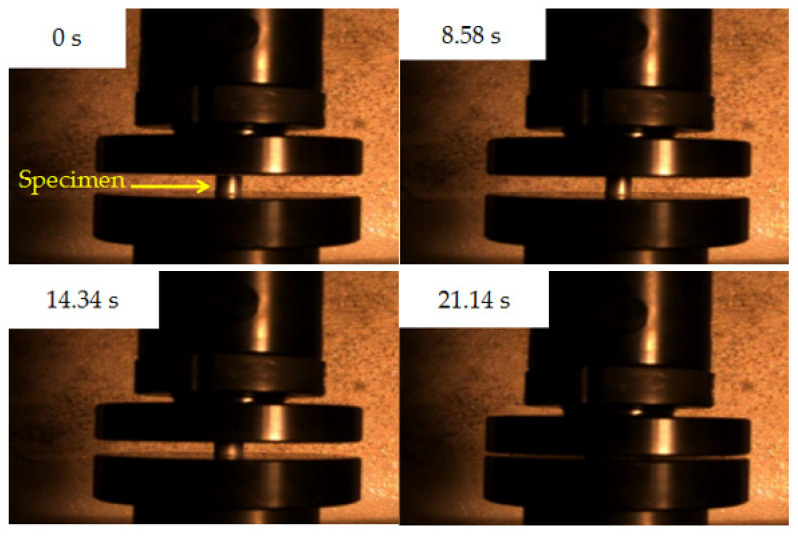
Quasi-static compression process of specimen 5.

**Figure 11:**
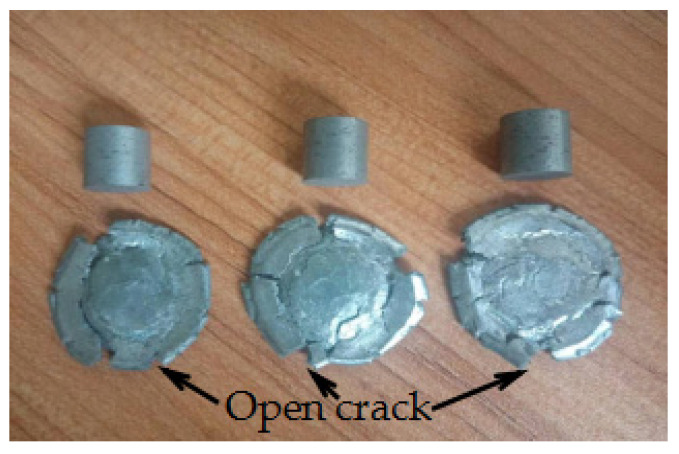
The residue of specimen 5 after three repeated compressions.

The quasi-static compression process of all the specimens was recorded with a high-speed camera at a speed of 50 fps. Figure 10 shows the compression process of Al-rich Al/PTFE/TiH_2_ active specimen 5 (240 MPa, 100 s) photographed using a high-speed camera. Figure 11 shows the residue of specimen 5 after three times of repeated compression. 

**Figure 12:**
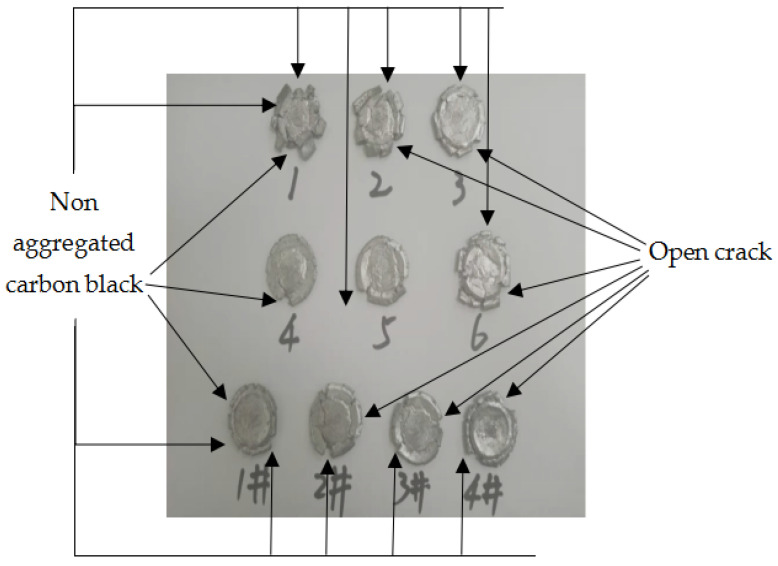
Residues after static compression experiment.

**Figure 13:**
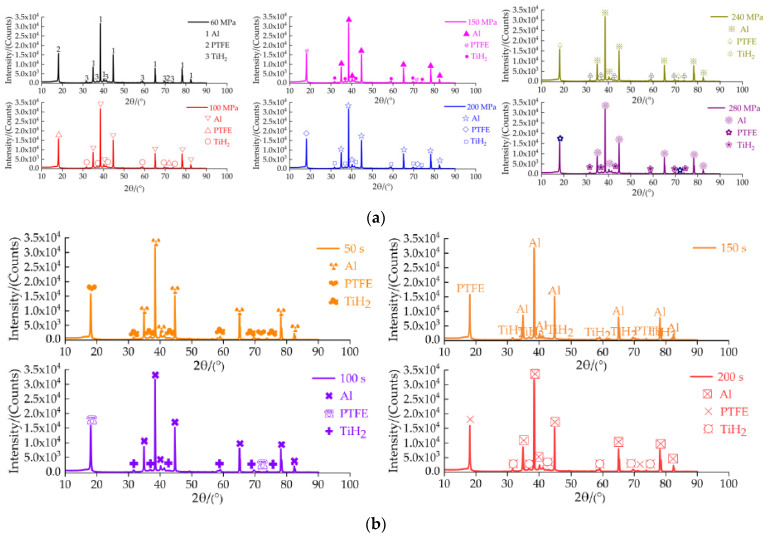
Phase analysis of residue after compression experiment was carried out. (**a**) XRD patterns of residues with different preforming pressure; (**b**) XRD patterns of residues with different the pressure holding time.

It can be seen from Figure 10 that the compression process of the specimen was completed from the beginning to 21.14 s, and no bright fire light was produced. It can be seen from Figure 11 that when the strain is 1.6711 and the failure stress is 76.672 MPa, specimen 5 begins to fail. When the strain continues to increase, shear microcracks are formed in the direction of the maximum longitudinal shear stress inside the specimen. These microcracks continue to expand and merge with the increase in strain, forming macro large and small open cracks. The specimen with high toughness absorbs more energy during the quasi-static compression process and converges at the crack tip, resulting in high temperature at the crack tip. It is known from the mechanism of crack induction reaction [29], that the reaction is more likely to occur at high temperature under the combined action of high Al content, high energy additive TiH_2_ and open crack. By observing residues 1–6 and 1#–4#, it is found that there is no accumulated carbon black at each opening crack, as shown in Figure 12. As shown in Figure 12, all specimens are fractured and failure destroyed. The analysis is as follows: One is that the outer wall of the specimen propagates along the radial direction, while the crack penetrating the specimen appears in the axial direction, which is caused by the radial tension caused by the axial compression of the material. When the radial tensile force is greater than the tensile limit of the material, microcracks are formed. With the increase in strain, the cracks continue to expand and converge, and finally a macro axial open crack is formed at the edge. Second, in the process of active material specimen preparation, the combination of PTFE matrix and particles is not tight. When the external force acts on the specimen, when the external force acts on the specimen, the contact between particles is prone to dislocation, resulting in stress concentration, where the energy generated exceeds the energy required for microcrack nucleation, forming microcrack nucleation. After nucleation, the microcracks continue to expand and converge and, finally, forming the open crack at the edge of the specimen. It was proved that the Al/PTFE active sample with TiH_2_ content of 10% did not react under the excess of Al, but it reacted at zero oxygen balance [3]. Therefore, the coordination of the mass ratio of excess Al and TiH_2_ is the key factor affecting the reaction of Al-rich Al/PTFE/TiH_2_ active materials under static loading. In order to further verify whether the Al-rich Al/PTFE/TiH_2_ specimens reacts, the phase analysis (XRD) of the residue after compression experiment is carried out, as shown in Figure 13. Figure 13a shows the XRD patterns of residues with different preforming pressure, and Figure 13b shows the XRD patterns of residues with different the pressure holding time.

It can be found from Figure 13a,b that, whether from the preforming pressure of 60 MPa to 280 MPa, or from the pressure holding time 50 s to 200 s, the XRD patterns of all the residues are the same, and there are only diffraction peaks of Al, PTFE and TiH_2_ in the XRD patterns, but no diffraction peaks of other substances. It shows that the composition of Al-rich Al/PTFE/TiH_2_ residues is the same and no reaction occurs. Thus, it is proved that Al-rich Al/PTFE/TiH_2_ specimens do not react under static compression. Furthermore, excess Al may be the key factor affecting the non-reaction of Al-rich Al/PTFE/TiH_2_ under static loading. Therefore, the reactivity of Al-rich Al/PTFE/TiH_2_ specimens with TiH_2_ the content of 10% under quasi-static compression is not significantly affected by the compression parameters. 

## 4. Conclusions

In this paper, the mechanical properties and micromorphology of Al-rich Al/PTFE/TiH_2_ specimens under molding parameters were studied by means of universal material testing machine and scanning electron microscope. X-ray diffractometer was used to analyze the phase of the residue after quasi-static compression experiment. The conclusions can be drawn as follows:

1. Unsintered: with the increase in preforming pressure, the particles deform from point contact to surface contact, the contact area increases gradually, and the shape and orientation of voids are very irregular. When the preforming pressure is less than 150 MPa, the porosity of the specimen increases slowly at first and then decreases. When the preforming pressure is greater than 150 MPa, the porosity of the specimen increases first and then decreases. When the pressure holding time is no more than 100 s, the porosity of the specimen decreases gradually. When the pressure holding time is more than 100 s, the porosity of the specimen increases first and then decreases.
2. Sintering: when the preforming pressure is less than 100 MPa, the porosity of the specimen decreases gradually. When the preforming pressure is greater than 100 MPa, the porosity of the specimen first increases and then decreases. With the increase in the pressure holding time, the porosity first increases and then decreases. For each preforming pressure specimen, compared with that before sintering, the porosity after sintering either decreases or increases. For each the pressure holding time specimen, the porosity increases after sintering compared with that before sintering. The microstructure of PTFE crystal in the specimen is mainly planar PTFE crystal. The size and number of planar PTFE crystals are significantly affected by the molding parameters. When the preforming pressure is less than 100 MPa, the planar PTFE crystals are small and less, the aggregates between PTFE matrix and particles are large and small, most particles are exposed, and the interfacial strength between particles is the lowest, which results in the worst mechanical properties of the specimens. When the preforming pressure is greater than 100 MPa and does not contain 240 MPa, the planar PTFE crystals are smaller and more, the voids are larger and more, and the interfacial strength between particles is higher, which results in better mechanical properties of the specimens. When the pressure holding time is 100 s, the planar PTFE crystals are large and more, and the interfacial strength between particles is the highest, which resulting in the best mechanical properties of the specimen.
3. With the increase in preforming pressures (60 MPa, 100 MPa, 150 MPa, 200 MPa, 240 MPa, and 280 MPa), the compressive strength, failure strain and toughness of Al-rich Al/PTFE/TiH_2_ specimens first increase and then decrease. When the preforming pressure is lower than 240 MPa, the density of Al-rich Al/PTFE/TiH_2_ increases rapidly at first and then increases slowly. When the preforming pressure is not less than 240 MPa, the density of Al-rich Al/PTFE/TiH_2_ specimen is almost unchanged. When the preforming pressure is 240 MPa, the compressive strength of the specimen reaches the maximum, and its failure strain and toughness are better. When the preforming pressure is not less than 150 MPa, the maximum true strain of the specimen is more than 2, which indicates that the specimen with preforming pressure above 150 MPa has good ductility. Based on the above knowledge, the specimen with the preforming pressure of 240 MPa has the highest strength, better failure strain, better toughness and good ductility.
4. With the increase in the pressure holding time (50 s, 100 s, 150 s and 200 s), the density and failure strain of Al-rich Al/PTFE/TiH_2_ specimens decreased, and their compressive strength and toughness increased first and then decreased. When the pressure holding time is 100 s, the density and failure strain of specimen are at moderate level, and its strength and toughness are the largest. The maximum true strain of specimens with different the pressure holding time is more than two, which indicates that the specimens with different the pressure holding time have good ductility. Based on the above knowledge, the overall mechanical properties of the specimen with the pressure holding time of 100 s are the best.
5. After quasi-static compression experiment, there are no diffraction peaks of other substances in the XRD patterns of all Al-rich Al/PTFE/TiH_2_ residues, and only diffraction peaks of Al, PTFE and TiH_2_ exist. It shows that Al-rich Al/PTFE/TiH_2_ specimens with TiH_2_ the content of 10% has no reaction occurred under quasi-static compression. It can be inferred that excess Al may be the key factor affecting the non-reaction of Al-rich Al/PTFE/TiH_2_ active materials under static loading. Therefore, the reactivity of Al-rich Al/PTFE/TiH_2_ specimens with TiH_2_ the content of 10% under quasi-static compression is not significantly affected by molding parameters.

## Figures and Tables

**Table 1:** The physical and chemical properties of each component of Al-rich Al/PTFE/TiH_2_ active materials.

Component	Density/(g/cm^3^)	Appearance	Melting/Boiling Point (K)	Decomposition Point/(K)	Theoretical ∆*H* of Reaction with Al/(kJ/g)
PTFE	2.200	White powder	614/-	829	28.9
Al	2.702	Silver grey powder	933/2600	-	-
TiH_2_	3.910	Dark grey powder	673/-	973	-

**Table 2:** Parameters of Al-rich Al/PTFE/TiH_2_ active materials under different preforming pressures.

No.	The Preforming Pressure/(MPa)	The Pressure Holding Time/(s)	Density ^1^/(g/cm^3^)	Relative Density ^2^/(%)
1	60	100	2.167	87.028
2	100		2.315	92.972
3	150		2.397	96.265
4	200		2.404	96.546
5	240		2.428	97.510
6	280		2.427	97.470

^1^ Density can be obtained from equation (6); ^2^ Relative density can be obtained from Equations (3) and (6).

**Table 3:** Static compression mechanical properties of Al-rich Al/PTFE/TiH_2_ active materials under different preforming pressures.

No.	Compressive Strength/(MPa)	Failure Strain	Toughness/(MJ/m^3^)
1	40.777	0.4337	16.638
2	50.114	1.0458	43.961
3	68.395	1.8878	92.213
4	64.157	1.6705	73.441
5	76.672	1.6711	89.630
6	66.690	1.3160	68.131

**Table 4:** Experimental mass loss rate of Al-rich Al/PTFE/TiH_2_ specimen 1, 2, 3, 4, 5 and 6.

No.	Measured Residue Mass m/(g)	Experimental Mass Loss Rate ^3^ η/(%)
1	1.750	10.532
2	1.815	7.209
3	1.860	4.908
4	1.837	6.084
5	1.865	4.652
6	1.843	5.777

^3^ The experimental mass loss rate η can be obtained by Equation (9).

**Table 5:** Parameters of Al-rich Al/PTFE/TiH_2_ active materials with different the pressure holding time.

No.	The Preforming Pressure/(MPa)	The Pressure Holding Time/(s)	Density ^4^/(g/cm^3^)	Relative Density ^5^/(%)
1#	240	50	2.430	97.590
2#		100	2.428	97.510
3#		150	2.423	97.309
4#		200	2.397	96.265

^4^ Density can be obtained from Equation (6); ^5^ Relative density can be obtained from Equations (3) and (6).

**Table 6:** Experimental mass loss rate of Al-rich Al/PTFE/TiH_2_ specimen 1#, 2#, 3# and 4#.

No.	Measured Residue Mass m/(g)	Experimental Mass Loss Rate ^4^ η/(%)
1#	1.650	15.644
2#	1.865	4.652
3#	1.763	9.867
4#	1.608	17.791

^4^ The experimental mass loss rate η can be obtained by Equation (9).
